# National Association of Dental Faculties

**Published:** 1892-08

**Authors:** 


### NATIONAL ASSOCIATION OF DENTAL FACULTIES.

The ninth annual meeting of the National Association of Dental Faculties was held at the Cataract House, Niagara Falls, commencing Monday, August 1, 1892.

Twenty-six colleges were represented, as follows:

Baltimore College of Dental Surgery—R. B. Winder.

Boston Dental College—J. A. Follett.

Chicago College of Dental Surgery—Truman W. Brophy.

Harvard University, Dental Department—Thomas Fillebrown.

Kansas City Dental College—J. D. Patterson.

Missouri Dental College, Dental Department of Washington University—W. H. Eames.

New York College of Dentistry—Frank Abbott.

Ohio College of Dental Surgery—H. A. Smith.

Pennsylvania College of Dental Surgery—C. N. Peirce.

Philadelphia Dental College—J. E. Garretson.

University of Iowa, Dental Department—A. O. Hunt.

University of Michigan, Dental Department—J. Taft.

University of Pennsylvania, Dental Department—James Truman.

Vanderbilt University, Dental Department.—W. H. Morgan.

Northwestern College of Dental Surgery—B. J. Roberts.

Louisville College of Dentistry—Francis Peabody.

Indiana Dental College—J. E. Cravens.

Northwestern University Dental School—E. D. Swain.

Dental Department of Southern Medical College—William Crenshaw.

Dental Department of University of Tennessee—J. P. Gray.

School of Dentistry of Meharry Medical Department of Central Tennessee College—G. W. Hubbard.

University of Maryland, Dental Department—John C. Uhler.

Columbian University, Dental Department—H. C. Thompson.

Royal College of Dental Surgeons of Ontario—J. Branston Willmott.

American College of Dental Surgery—John S. Marshall.

University of Denver, Dental Department—George J. Hartung.

The *ad interim* committee reported that it had investigated a charge preferred against the University of Maryland, Dental Department, by the College of Dentistry of the University of California, of graduating a person in less time than the rules demanded; that it found that no rule of the association had been violated, and had so reported to the parties in interest; that it had dismissed an effort for the reinstatement of the American College of Dental Surgery, Chicago, as not within the jurisdiction of the committee, with the advice to reorganize the college before attempting to influence the association to change its action, which reorganization has since been accomplished.

The committee also stated that its value in settling such matters had been made so clearly apparent that it recommended that it should be made a standing committee, to be elected by the association, instead of being appointed by the president.

The report was received and placed on file, and the recommendation with regard to the status of the committee was adopted.

The following resolutions, laid over from last year, were adopted:

*Resolved*, That in case of charges against any college no final action shall be taken until all parties concerned shall have at least thirty days' notice.

*Resolved*, That at all future meetings of the National Association of Dental Faculties the delegates shall consist of members of faculties, and demonstrators will not be received.

The following resolutions, also over from last year, were laid on the table:

*Resolved*, That after June, 1893, the yearly course of study shall be not less than seven months, two months of which may be attendance upon clinical instruction in the infirmary of the school, now known as intermediate or infirmary courses.

*Resolved*, That after the session of 1892 93, tour years in the study of dentistry be required before graduation.

The following resolutions lie over under the rules:

Offered by Dr. Winder,—

*Resolved*, That hereafter graduates of pharmacy be placed on the same footing as graduates of medicine, and be entitled to enter the second-year or junior class, subject to the examination requirements of each college.

Offered by the executive committee,—

Any college failing to have a representative present for two successive sessions without satisfactory explanation shall be dropped from the roll of membership of this association.

The chair, having been asked for a ruling upon the admission of graduates of pharmacy to the junior class, decided that under the rules they could only be admitted to the first-year or freshman class.

The executive committee offered a report recommending the restoration of the American College of Dental Surgery to full membership, which, after an explanation by Dr. Marshall of the reorganization of the college, was unanimously adopted.

The executive committee reported on the application of the Western Dental College, of Kansas City, recommending that it lie over for one year. The report was adopted.

The report of the executive committee recommending the rejection of the application of the Tennessee Medical College, Dental Department, of Knoxville, Tenn., for irregularities in conferring the degree of D. D. S. and in the reception of students, was adopted.

The application of Howard University, Dental Department, Washington, D. C., was laid over for one year.

The following applications for membership, also reported by the executive committee, lie over under the rules:

United States Dental College, Chicago.

Homœopathic Hospital College, Dental Department, Cleveland.

Detroit College of Medicine, Department of Dental Surgery.

The report of the executive committee recommending that the Baltimore College of Dental Surgery be censured by the association for conferring the degree of Doctor of Dental Surgery upon Charles F. Forsham, M. A., LL. D., of Bradford, England, *in absentia* and honorarily, in violation of the rules of the association, was adopted.

Dr. Truman offered an amendment to the rule regarding the conferring of the degree of Doctor of Dental Surgery honorarily, absolutely prohibiting the exercise of that privilege to the members of the association, but the amendment was lost, after discussion, it being the general sense that the present rule is a sufficient safeguard against the unworthy bestowal of the honor.

Dr. Cravens offered the following amendment to the constitution, which goes over under the rules:

Amend Article VII. so that it shall read as follows:

ART. VII. Any reputable dental college, located in any State of the United States, may be represented in this body upon submitting to the executive committee satisfactory credentials, signing the constitution, conforming to the rules and regulations of this body, and paying such assessments as may be made.

The association adopted a protest against the classification of dentists as manufacturers, as provided in House Bill No. 7696, known as the Willcox bill, and against the collection of statistics from dentists under its provisions, on the grounds that dentists are not manufacturers in any sense, not being engaged in the manufacture, fabrication, or sale of any product having a merchandisable value; that all the laws heretofore passed in the various States and Territories and the District of Columbia distinctly recognize dentists as professional men; and that the attempt to collect statistics would be an injustice not only to them but to their patients, and that such statistics if collected would be valueless to the government because showing the products of a class of men not engaged in manufactures.

The following, offered by Dr. Winder, was also adopted:

*Resolved.* That the National Association of Dental Faculties recommends that their alumni write and demand of the Census Bureau of the United States the return of all statistical reports, as, under the recent agreement between the dental profession and said Bureau, lawyers, physicians, and dentists are exempted from making statistical reports for the census of 1890, and that a copy of this resolution be forwarded to the chief of the Census Bureau.

A communication from the Post-Graduate Dental Association of the United States, suggesting the establishment by the colleges of short courses of training and teaching especially designed and arranged for practitioners, was received and referred to the executive committee.

The manuscript of a Compend of Materia Medica and

Pharmacy for Dental Students, by Dr. E. L. Clifford, of Chicago, was referred to the committee on text-books, with power to act.

Dr. Marshall offered the following resolution, which was adopted:

*Resolved*, That the secretary be instructed to notify the National Association of Dental Examiners that the National Association of Dental Faculties considers it out of its province to legislate upon the relative values of the L. D. S. and D. D. S. degrees.

The following were elected officers for the ensuing year: J. D. Patterson, Kansas City, president; H. A. Smith, Cincinnati, vice-president; J. E. Cravens, Indianapolis, secretary, H. A. Smith, Cincinnati, treasurer; F. Abbott of New York, J. Taft of Cincinnati, and A. O. Hunt of Iowa City, executive committee; James Truman of Philadelphia, Frank Abbott of New York, and Thomas Fillebrown of Boston, *ad intertm* committee.

The president appointed as the committee on schools Drs. J. A. Follett, Boston; S. H. Guilford, Philadelphia; E. D. Swain, Chicago; C. N. Peirce, Philadelphia; T. W. Brophy, Chicago.

Adjourned to meet at the call of the executive committee.

